# Focal myocardial effects in infective endocarditis

**DOI:** 10.1007/s12471-022-01721-8

**Published:** 2022-09-08

**Authors:** V. A. W. M. Umans, Tj. Germans, M. G. J. Duffels

**Affiliations:** Department of Cardiology, Noordwest Ziekenhuisgroep, Alkmaar, The Netherlands

A 72-year-old male presented to the emergency department with syncope, malaise and recent episodes of cold chills. His medical history included severe mitral valve insufficiency and paroxysmal atrial fibrillation (AF) for which he received apixaban treatment in another hospital. On physical examination, his temperature was 37.9 °C, his heart rate was irregular at 118 bpm, his blood pressure was 123/72 mm Hg, and a cardiac holosystolic murmur was heard at the apex and in the axilla. The abdomen was normal, and one nail showed a splinter haemorrhage.

Electrocardiograms showed permanent AF without new ST‑T changes. Laboratory results revealed elevated infection parameters (C-reactive protein: 205 mg/l; leucocytes: 13.4 × 10^9^/l) and increased renal function and liver enzyme values. Repeated high-sensitivity (hs) troponin I levels were 1136, 1823 and 2265 ng/l. At admission, the haemoglobin level was 7.4 mmol/l and blood cultures were positive for *Streptococcus oralis*. Echocardiography showed a mildly dilated left ventricle with hypokinesia of the anteroseptal wall and a severe mitral valve insufficiency due to prolapse. Transoesophageal echocardiography revealed a mobile structure on the posterior mitral valve leaflet suspect of vegetation (Fig. 1).

**Fig. 1:**
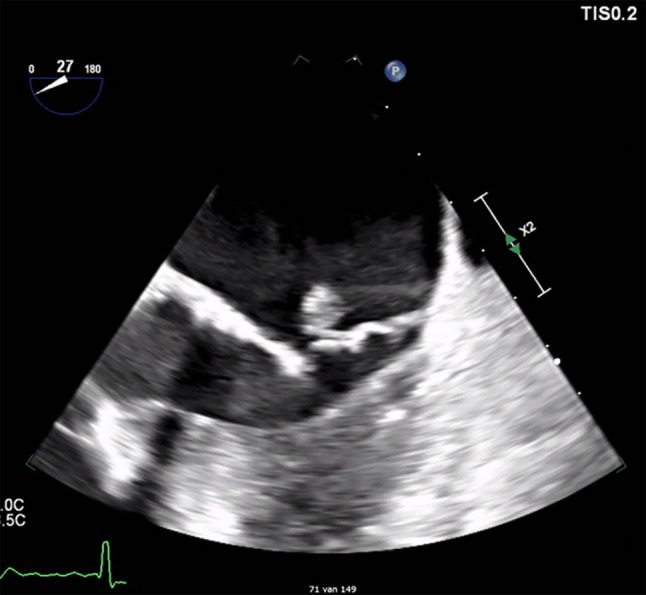
Transoesophageal echocardiographic image of mitral valve with vegetation on posterior valve leaflet

The asymptomatic hs–troponin I release prompted us to consider a working diagnosis along the four principle pathophysiological mechanisms of coronary obstruction: endothelial injury, hypercoagulability, blood stasis/low flow or anatomical predisposition [1–3]. A primary coronary event was unlikely given a recent normal coronary angiogram, AF-related embolism could be ruled out by adequate anticoagulation and a clear atrial appendage; no signs of hypercoagulability were found.

Which imaging modality would you choose to make the diagnosis?

## Answer

You will find the answer elsewhere in this issue.
